# Examining Healthcare Workers’ Perspectives Concerning Medical Equipment Availability in Three Ethiopian Hospitals: A Qualitative Pilot Study

**DOI:** 10.7759/cureus.14134

**Published:** 2021-03-26

**Authors:** Eyasu Kebede, Ezra Yoseph, Henock Asaye, Binyam Mulate

**Affiliations:** 1 Co-Founder, Oasis Medical Relief, Las Vegas, USA; 2 Program of Human Biology, Stanford University, Stanford, USA; 3 Department of Mother-Child Health, Nifas Silke Lafto Sub-City Worede 12 Health Center, Addis Ababa, ETH

**Keywords:** medical equipment, qualitative research, ethiopia, donations, distribution

## Abstract

Background

Hospitals in the United States often have an abundance of unused medical supplies and equipment while many developing countries are in considerable need of these resources. Many nongovernmental organizations (NGOs) have donated medical equipment to health centers in low-resource settings to rectify this issue; however, studies show many of these donations are not usable by the facilities that receive them. To better serve the partner hospitals of our NGO, Oasis Medical Relief, we investigated the perspectives and insights of Ethiopian healthcare workers (HCWs) on the medical equipment distribution paradigm of the country.

Methodology

Qualitative analysis including semi-structured, open-ended interviews was conducted. Semi-structured interviews (n = six) were conducted with HCWs (four physicians and two hospital administrators) working in hospitals in Addis Ababa and Southern Nations, Nationalities, and Peoples’ Region (SNNPR) of Ethiopia. Interviews were recorded and transcribed. Categorical content analysis was utilized to develop themes. The topical areas addressed by our questions include populations served, prevalence of diseases, laws, and strategies guiding medical equipment distribution, funding and budget for medical equipment, etc.

Results

Three themes related to perspectives and insights of HCWs on the current medical equipment distribution paradigm in Ethiopia interviewed include: (1) state of healthcare concerns, (2) medical equipment scarcity, and (3) policy shaping medical distribution paradigm.

Conclusions

Pre-donation assessments utilized to understand equipment needs are recognized by the World Health Organization to more effectively address medical equipment/supply. However, to further strengthen such efforts, qualitative interviews with HCWs are a tool that can be utilized to better understand the intricacies of Ethiopia’s complex medical distribution paradigm. This can potentially lead to more effective partnerships between NGOs and their partner hospitals. Furthermore, increasing decentralized methods of procuring medical equipment should be further explored to mitigate issues with national distribution of medical supplies.

## Introduction

Medical waste in the United States has been an issue for several decades, with healthcare facilities being responsible for approximately 4 million tons of medical waste each year. The majority of medical waste in healthcare facilities is typically found in the operating room [1]. Ever since single-use devices became common in the 1970s, medical waste in the United States has been increasing at an alarming rate [2-4]. There have been some efforts to address this issue at the federal and state level with varying levels of success [5]. At the same time, in the developing world, there is a great shortage of medical supplies and equipment [6-8]. The ramifications of this problem are amplified by the lack of proper medical infrastructure in a vast majority of developing nations [9].

It is abundantly clear that low-income countries face grave shortages of medical equipment and rely on foreign donations to sustain their healthcare system [6-8]. As such, there have been many efforts by nongovernmental organizations (NGOs) to donate much-needed medical equipment to bridge the gap between the developed and developing world. Although medical equipment donations to low-resource settings have some merit, the World Health Organization (WHO) estimates that at least half of the medical equipment donated to low-income countries is unusable. Many factors contribute to this, with the key components being a shortage of biomedical technicians in developing nations as well as ill-regard to the true needs of the recipient hospital staff [10].

In Ethiopia, significant reforms have been made in the healthcare infrastructure over the past few decades. This has led to improved healthcare outcomes. Since 1990, maternal and child mortality have decreased by 50% and 60%, respectively [11]. However, according to the WHO’s efficiency rating used to measure overall health system performance, Ethiopia ranks 180th out of the 191 countries surveyed [12]. The shortage of basic medical equipment accounts, in part, for the poor performance of the Ethiopian healthcare system. As an example, a 2014 study revealed that only 75% of general outpatient services of health facilities had thermometers and stethoscopes. At Health posts, which are small health facilities in Ethiopia serving rural communities, only 66% had the aforementioned supplies available. Only 48% of health facilities had child scales in their outpatient area [13]. Furthermore, in Ethiopian public hospitals, a WHO study showed that there are only two magnetic resonance imaging (MRI) machines and 12 computerized tomography (CT) scanners per 1,000,000 people [14].

The goal of this study was to examine the attitudes of healthcare workers (HCWs) in Ethiopia regarding medical equipment availability and utilization in the nation. An understanding of the key barriers faced by HCWs in recipient hospitals will bolster the impact of medical donations in the future and introduce more sustainable solutions to improve healthcare infrastructure in under-resourced regions.

## Materials and methods

Study design and sampling

**Figure 1:**
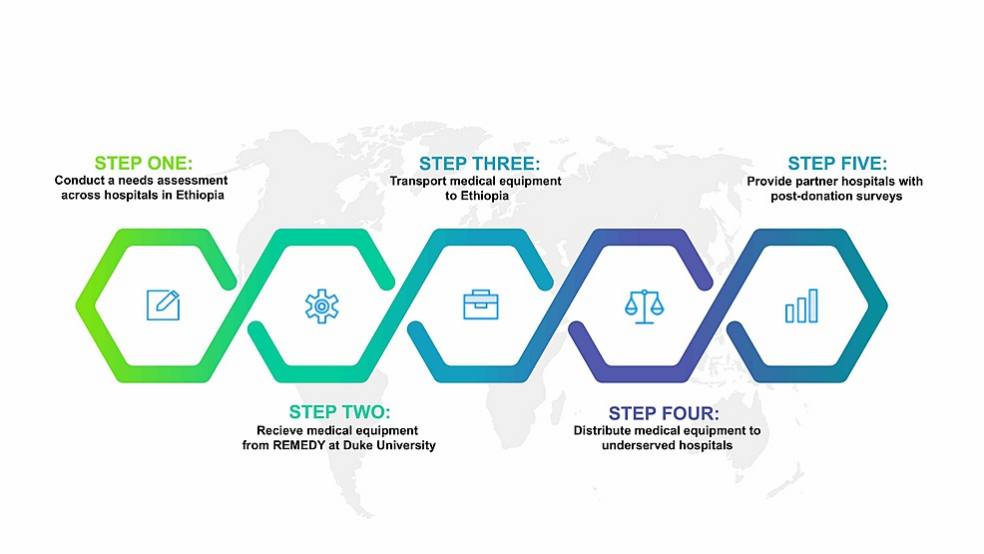
Oasis Medical Relief distribution schematic.

A qualitative and descriptive study was conducted in three governmental hospitals in Ethiopia. Two of these hospitals received medical equipment donations from our NGO, Oasis Medical Relief (OMR). A third, well-resourced hospital was included as a comparison. An outline of OMR’s distribution model is depicted in Figure 1. After conducting a pre-donation assessment of need, we found that our partner hospitals were in dire need of respiratory care equipment and basic medical supplies. We partnered with Recovered Medical Equipment for the Developing World (REMEDY) at Duke Healthcare to secure necessary equipment [15]. In the summer months of 2018, 2019, and January 2021, OMR donated gloves, surgical masks, nebulizer tubing, incentive spirometers, and other supplies to our partner hospitals (Appendix A).

Data collection and analysis

Study investigator (HA) conducted semi-structured qualitative interviews with six participants from the three target hospitals from May 6 to June 30, 2018 and from May 6 to 27, 2019. Interviews lasted from 30 to 45 minutes. While there was a list of prepared questions dealing with medical inequities and health policy, HCWs had the opportunity to ask additional questions for clarification and to obtain a broader understanding. Informed verbal consent was gathered from the HCWs; this research study design was reviewed and approved by the Duke University Institutional Review Board (Protocol #2018-0447). Participants included four physicians and two hospital administrators and were chosen based on their history of clinical practice and managerial duties. Thematic saturation was reached when no new categories or themes arose after interviewing five participants. Although all questions were asked in English, interviewees either responded in English or Amharic (Ethiopian official language) based on personal preference.

Every interview was audio recorded by study investigator and subsequently translated and transcribed by a third party, a commercial translating service. Thematic analysis and inductive reasoning were employed. While the research questions guided the focus of the analysis, additional themes raised from the interviews were included in the analysis. Emergent themes were realized based on using categorical codes and re-classifying raw data into those categories.

## Results

Participants included clinicians and healthcare administrators from three hospitals in Ethiopia (Table 1). The themes from interviewee responses included: state of healthcare concerns, medical equipment scarcity, and policies shaping the medical distribution paradigm. Quotations have been included to supplement descriptions of HCWs’ interviews.

**Table 1:** Demographics of healthcare workers. SNNPR, Southern Nations, Nationalities, and Peoples’ Region

Participant ID	Sex	Position	Areas served
001	Female	Physician	Addis Ababa
002	Female	Physician	SNNPR
003	Male	Physician	SNNPR
004	Male	Medical Director	SNNPR
005	Male	Finance Administrator	SNNPR
006	Male	Physician	Addis Ababa

State of healthcare concerns

In their patient pool, HCWs cited tuberculosis (TB) and other respiratory infections as the most prevalent communicable diseases and hypertension, renal, and cardiovascular diseases as most prevalent noncommunicable diseases. To mitigate negative health outcomes in Ethiopia, an HCW mentioned decreasing smoking and junk food intake to be high priority public health efforts. In order to better address patient population’s needs, one HCW mentioned the importance of properly equipping hospitals instead of simply building new hospitals:

“...instead of building different hospitals all over the place, we should be able to see which places have the larger population...and be able to equip those hospitals instead of randomly putting health centers there without equipment...” (002).

A mission of the partner hospitals was to improve pregnancy and the birthing experiences for women. HCWs indicated the importance of recommending prenatal care that includes regular check-ups for pregnant women. HCWs also recommended an effort to increase hospital births and drive down the incidence of at-home births. As a result, prenatal and postnatal care for women is free, and they are only required to pay for their transportation. To tackle issues like postpartum bleeding or birth-related infections, this hospital utilized health-extension workers (community health educators native to the area they serve) to promote proper medical care.

Other issues brought up during the interviews included concerns regarding hospital staff shortages and high employee turnover rates which forced hospitals to hire part-time HCWs.

Medical equipment scarcity

HCWs purported that a governmental organization known as “Ethiopian Pharmaceuticals Supply Agency” (EPSA) (formerly known as the PFSA) is the largest importer of medical equipment and medicine in Ethiopia tasked with supplying the country’s government hospitals. EPSA is also responsible for allocating the aforementioned resources to healthcare facilities countrywide. Yet, HCWs reported that shortage of medical equipment, medicine, and lab reagents is evident across several hospitals in the country. Furthermore, the time it takes to secure requisite resources remains rather lengthy:

“Right now, getting medical equipment could take a hospital 6 or 7 months...” (001).

Shortages are exacerbated when considering the urban-rural divide due to the sparsity of healthcare facilities in rural areas. Urban areas face unique healthcare challenges as well. HCWs reported that the high-density populations in urban areas makes equity of care challenging.

HCWs cited a limited supply of respiratory care equipment, such as oxygen cylinders, pulse oximeters, and concentrators. For this reason, they reported using their clinical judgment to decide which patients get access to medical equipment. One HCW related an anecdote of a patient death during surgery due to insufficient availability of proper medical equipment:

“About 3 weeks ago, I had a patient who sustained a road traffic accident...I was about to insert a chest tube...we were searching for the equipment and we lost her immediately” (003).

If there is a shortage of beds, clinicians mentioned that patients must be referred to other hospitals free of charge as many cannot afford to be treated in private hospitals. Despite broad-spectrum antibiotics being widely available, clinicians specified that there were very few potent antibiotics available to treat more complex cases due to costs. At one surveyed hospital, the nominal intensive care unit (ICU) is not equipped with critical care equipment. Though they planned to revamp their ICU to provide prenatal and neonatal care, their efforts have not yet been successful in large part due budget shortfalls:

“We have patients with respiratory failure despite getting the most potent antibiotics we have. In that case, we need more respiratory equipment and these are a problem not just here but generally in the country in ICU setups...” (002).

The inadequate supply of resources results in clinicians rationing supplies necessary to treat their patients.

Policies shaping medical distribution paradigm

When describing the organizational framework of Ethiopia’s healthcare system, HCWs cited Ethiopia’s healthcare landscape is analogous to the country’s governing structure, where regions are divided up into zones and woredas (districts). At the top of the hierarchy lies the Federal Ministry of Health (FMoH). Hospitals receive funds from the FMoH and use it to compensate medical and non-medical personnel. HCWs described the following: If a hospital does not report spending all of its funds, it will receive less in the next fiscal year. The regional bureau approves or denies medical equipment and medicine purchasing requests from hospitals, and this approval process occurs through bidding. An HCW also mentioned hospitals can secure minimal funding from the government to buy items such as vaccines from private companies, accessing a maximum of 250,000 Ethiopian birr (approximately 6,000 USD). Moreover, HCWs reported inefficiencies in the government’s handling of the medical equipment distribution:

“To my understanding, PFSA is the largest importer of medical equipment and medicines in Ethiopia. There is also some private sector involvement that hospitals can utilize, as needed” (006).

Although they assert the government has begun to prioritize health as a central issue, they believe responses to health crises to be particularly inadequate. Regular communications at health conferences, including meetings between HCWs and the Ethiopian Prime Minister, have rekindled a new sense of hope among Ethiopian healthcare professionals. Overall, recommendations of HCWs included improving medical equipment by diversification and potential privatization of medical equipment allocation and reducing the EPSA monopoly.

## Discussion

State of healthcare concerns

HCWs recognized that cardiovascular diseases are a major concern for providers in Ethiopia. This is evidenced by the Global Burden of Disease Study, which attributes cardiovascular diseases as the leading cause of mortality in the country [16]. Furthermore, as TB remains to be a major health concern in Ethiopia, in 2008, it was found that 43% of men with active TB disease were smokers, with a male population incidence of smoking of 8.1% [17,18]. The prevalence of these conditions is informative as they dictate the types of medical equipment most needed at these institutions and the country more broadly.

With regards to our partner hospital’s efforts to decrease at-home births through the use of health extension workers, particularly in rural areas, it is still not commonplace to give births in health centers unless women face complications during birth. To combat this, there has been a nationwide effort to use health extension workers to better understand the barriers to increasing hospital births. One qualitative study found distance, lack of transportation, and sociocultural factors as main drivers for why women did not go to healthcare facilities to give birth [19]. These are important parameters our partner hospital still needs to consider to maximize the efforts of their health extension workers.

Medical equipment scarcity

A prominent issue our HCWs detailed was the acquisition of medical equipment and medicine facing regular delay. One study showed that the average time it took to receive antiretroviral drugs was 46.4 days in hospitals and 59.2 days in Ethiopian health centers [20]. A study examining the availability of TB reagents found a similar result, where 40.2% of health centers surveyed did not have at least one of the key reagents needed for diagnosing patients with TB [21]. This issue is not unique to health centers in Ethiopia and is noted in many other underdeveloped regions.

In the past, medical donations have been a strategy to address the prevailing issue of medical equipment shortages, and pre-donation surveys have been recommended by the WHO as a tool to make donations more impactful. Although pre-donation surveys are a good first step to enhance the effectiveness of the donation process, as the end goal is to improve patient outcomes, we recommend a more comprehensive approach. Experts continue to find that surveys do not go far enough to properly assess concerns of stakeholders because the information reported cannot provide an exhaustive understanding of a partner hospital’s needs and concerns. Directly approaching and asking open-ended interviews, on the other hand, yields better strategy and implementation efforts [22]. Furthermore, the WHO states that a lack of genuine partnership between donors and recipients is a key barrier to effective medical equipment donation [23].

Typically, pre-donation surveys only capture the need as determined by the administrator tasked with filling out the survey. This approach may not capture the social and political issues that affect the hospital’s needs at a given time. A more intimate connection with partners is formed when varied viewpoints are presented through a semi-structured interview setting. To that end, we conducted in-depth interviews of multiple healthcare providers to give investigators a clearer understanding of the needs of partner hospitals in hopes of improving the donation process. In our own experience interviewing healthcare workers at an urban SNNPR hospital, we found that the HCWs were excellent sources of information on the paucity of medical equipment which affects their patient’s outcomes and overall quality of treatment. Interviewing the financial administrator, on the other hand, gave us insight into the amount of funding and medical equipment hospitals historically receive and how that relates to other regional hospitals. This financial administrator was well versed in the legislations set by Ethiopian regulatory authorities, and he described inconsistencies in the implementation of laws regarding governmental medical equipment distribution. By combining these two viewpoints, we were better able to understand and address patient-centered concerns while acknowledging certain underlying sociopolitical factors.

Connecting developing world hospitals with a medical surplus organization seeking to bring medical equipment donations in the developed world has also been a great impediment to making donations abroad. Currently, Yale REMEDY has a virtual warehouse initiative that allows nonprofit organizations to be connected to donors online, called Med-Eq. By utilizing this initiative, people interested in service projects can make a meaningful impact in communities abroad. In the same way, people interested in globally equitable distribution of medical equipment can and should partner with hospitals in the developed world to secure medical equipment. Once they have secured these partnerships with local hospitals, they must also consider maintaining equally strong relationships with their partner hospitals abroad. This has the potential to both alleviate medical waste in the United States and Europe as well as reduce medical equipment scarcity in low-resource settings.

Policies shaping medical distribution paradigm

Ethiopia’s regions are divided into zones, which are subdivided into woredas (districts) [24]. This organizational structure is informative of the nation’s healthcare landscape. Regarding the procurement process, EPSA uses need-based pooled procurement to acquire medicine and medical equipment [25]. Sentiments behind EPSA’s inadequate actions are shared by other healthcare professionals in Ethiopia, with one report stating that primary hospitals did not receive routine and life-saving medications that clinicians regularly prescribe [26]. Internal studies within EPSA found that having a reference list of medical equipment and medicine will enable them to track their progress more purposefully instead of trying to procure every medicine and equipment that healthcare facilities request. Though this reform is a step in the right direction, EPSA still supplies approximately 60% of all medical equipment and medicine in Ethiopia [27]. Therefore, it is imperative for the country to further diversify by including more private distributors to meet the vast demand for medical equipment and medicine.

The United Nations Commission on International Trade Law Model Law on Procurement of Goods, Construction and Services (UNCITRAL Model Law) was designed to assist countries in need of an efficient framework for regulating the procurement of medical equipment and medicine. Since 2010, many sub-Saharan African countries, such as Ghana, Kenya, Rwanda, and Tanzania, have adopted legislation in support of the UNCITRAL Model Law. Though a central authority is recommended to facilitate domestic regulation, a more decentralized means of procuring the equipment is advised [28]. Therefore, we highly recommend EPSA continues its role as a regulatory body while promoting more diversification in the procurement processes. As healthcare professionals continue discourse between the private and public health sectors, improvements such as this will bolster medical infrastructures and health policy in Ethiopia to ensure equitable medical distribution.

Limitations

This study had a small sample size and was broad in scope. Though the sample size was enough to reach thematic saturation, our participants only included individuals from the capital city (Addis Ababa) and the SNNPR region. Therefore, a full picture of the healthcare system in Ethiopia was not represented, leading to selection bias. A true firsthand account of nuances in rural areas was also not gained. A further review of the summary interview guide also revealed some questions that may have been framed in ways that introduced bias. Moreover, due to the nature of qualitative research, replicability is difficult.

## Conclusions

When it comes to the distribution of medical equipment, our study revealed that qualitative interviews are an asset in building strong and sustainable partnerships with hospitals in underserved areas. Moreover, centralization of distribution hinders the formation of efficient distribution systems involving increased privatization of medical equipment procurement. We strongly recommend utilizing a multi-pronged approach for service projects. Based on findings from this study, future studies that examine inefficiencies of EPSA are important to consider. In doing so, they could potentially identify failures that EPSA may consider reforming through both internal and external audits. A continued examination of hospital-specific issues to better administer the sustainable implementation of medical equipment donation is also recommended. Furthermore, future interviews with a larger sample and in additional urban and rural areas in Ethiopia are important to consider for investigation. Additional research could be conducted regarding the training capabilities of biomedical technicians with the aim of salvaging medical equipment that is faulty and needs repair. In closing, there remain several opportunities to investigate bottlenecks in medical equipment distribution impacting developing countries.

## Appendices

Appendix A

List of donated medical supplies/equipment

Basic supplies

Thermometer probe covers

Gloves

Surgical masks

Hot packs

Analgesic medications

Respiratory 

Tubings - oxygen

Nebulizer tubings

Small-volume nebulizers

Anesthesia breathing circuits

Incentive spirometers

Drapes

Laboratory drapes

Plastic drapes

Poly-lined sterile field drape

Cardiology 

Red dot monitoring electrodes

Stethoscopes

Catheter

Catheter bag

Catheters

Thoracic catheters

Suture and skin closure

Skin closure strips

Wound suture kits

Miscellaneous

DNA kits

Canister for gel therapy

Urinary drainage bags
